# Cystic adenomyoma of the uterus: Case report and literature review

**DOI:** 10.1515/biol-2022-0846

**Published:** 2024-05-15

**Authors:** Lei Zhang, Zhaojun Guo, Yicun Pang, Jun Zhao, Jun Liang, Xiaolin Ma

**Affiliations:** Department of Obstetrics and Gynecology, The Third Hospital of Hebei Medical University, No.139 of Ziqiang Road, Qiaoxi District, Shijiazhuang, Hebei Province 050051, China

**Keywords:** cystic adenomyoma of the uterus, dysmenorrhoea, diagnosis, surgery

## Abstract

Cystic adenomyosis is a rare type of adenomyosis. The main clinical manifestation of uterine cystic adenomyoma is severe dysmenorrhoea, and the condition can be diagnosed by relevant clinical examination. The preferred treatment, with a good prognosis, is lesion resection. The clinical data of a patient with uterine cystic adenomyoma recorded at the Third Hospital of Hebei Medical University are reported herein. A 39-year-old female patient presented with tolerable menstrual pain and aggravated dysmenorrhoea, which she had experienced for 4 years, and menorrhagia, which she had had for approximately 1 year. Ultrasound and tumour marker tests suggested abnormalities, and magnetic resonance imaging confirmed a diagnosis of uterine cystic adenomyoma. A hysteroscopy and intrauterine lesion electrocision were performed, and the results of postoperative pathology tests suggested that the endometriosis cysts had returned to normal after the postoperative intervention. The analysis of the clinical manifestations and diagnosis and treatment of uterine cystic adenomyoma can improve the understanding of the disease and reduce the rates of misdiagnosis and missed diagnoses to ensure early detection with timely diagnosis and treatment.

## 1 Introduction

Adenomyosis is a benign uterine disease characterised by gland and stromal tissue of the ectopic endometrium invading the myometrium with reactive fibrosis [1]. If the cystic cavity is filled with ectopic endometrial tissue and blood with a diameter of greater than 10 mm, the condition is referred to as cystic adenomyosis, an adenomyotic cyst, or cystic adenomyoma [2]. This is a rare type of adenomyosis with low incidence, and its typical clinical manifestations are dysmenorrhoea and menorrhagia, which are difficult to control. Asymptomatic cases are usually not diagnosed in routine gynaecological examinations; however, some patients with cystic adenomyosis complain of severe pain associated with menstruation [3]. In other cases, it may be found during the investigation of infertility or miscarriage. This disease has a negative impact on quality of life in terms of menstrual symptoms, fertility, and pregnancy outcomes, and patients are at high risk of miscarriage and obstetric complications [4]. Cystic adenomyoma is still a poorly understood disease. It is a cystic lesion that does not communicate with the uterine cavity. It is lined with a healthy endometrium, lies within the myometrium, and contains a bleeding fluid described as chocolate like. According to the literature, patients with symptomatic cystic adenomyoma present mainly with pain, especially dysmenorrhoea, sometimes accompanied by chronic pelvic pain and painful intercourse. Menorrhagia and menorrhagia are sometimes associated with cystic adenomyoma. Few cases of cystic adenomyoma have been reported in the literature. Similar disease entities have been reported using different nomenclature, making it difficult to estimate the prevalence of the disease. Most case reports show that the lesion site of uterine cystic adenomyosis is often located in the myometrial wall or subserosa. Due to the lack of specificity of clinical manifestations, it is easy to miss a diagnosis of uterine cystic adenomyosis or misdiagnose it before surgery, and since an early diagnosis is difficult, this condition has attracted much attention. This article presents a clinical case of cystic adenomyoma in a 39-year-old woman who reported menstrual abdominal pain and summarises the patient’s clinical manifestations, classification, pathogenesis, diagnosis, and treatment to provide a reference for clinicians to diagnose and treat this disorder.

## 2 Case description

### 2.1 History and pre-admission examination

The 39-year-old female patient was hospitalised on 23 February 2021 due to dysmenorrhoea, which she had experienced for 4 years, and aggravation of menstruation, with increased menstruation volume and length, which she had had for 1 year. The patient had had four pregnancies but only one birth, a male baby 13 years previously, who had been delivered by caesarean section. The section incision had healed poorly, and the patient had fat liquefaction, infection, incision dehiscence, and purulent secretion exudation in the abdominal incision. The incision finally healed after repeated dressing changes, anti-infection treatment, and secondary suture. The patient had undergone two artificial abortions and a laparoscopic right salpingectomy, due to an ectopic pregnancy. The patient’s menstrual abdominal pain was tolerable and had a visual analogue scale (VAS) score of 2 points, and the aggravated dysmenorrhoea symptoms had a VAS score of 9 points. Oral analgesics administered daily during menstruation relieved the symptoms of dysmenorrhoea, but they increased menstrual flow and prolonged menstruation.

Ultrasound examination of the patient 2 months prior to admission showed uterine cavity line separation, a 59 mm × 24 mm liquid dark area, poor sound transmission, dense weak echo points, and unilateral endometrial thickness of approximately 2.3 mm. An intrauterine fluid dark area (a haematocele) was considered and curettage was suggested, but the patient refused it. On 16 February 2021, the patient visited another hospital and underwent a gynaecological ultrasound, which suggested that the low-echo area in the uterine cavity had dimensions of approximately 2.6 cm × 3.5 cm × 2.4 cm. As a result of this intrauterine low-echo area (a possible haematocele), a curettage was performed. No blood was discharged during the operation, and only a small amount of endometrial tissue was scraped out. The pathological results indicated secretory changes in the endometrium, accompanied by decidual-like changes in the stroma.

After hospital admission, the patient underwent further clinical examinations to confirm the initial findings and make a diagnosis. Ultrasound and magnetic resonance imaging (MRI) imaging were performed to observe and locate the lesion, and the tumour markers carbohydrate antigen (CA125) and carbohydrate antigen 19-9 (CA199) were checked to further confirm and track the changes in the cystic adenomyosis. Finally, after the initial diagnosis, a hysteroscopy and intrauterine focal electrocision surgery were performed to remove the lesion, which was analysed using haematoxylin and eosin (HE) staining.


**Informed consent:** Informed consent has been obtained from all individuals included in this study.
**Ethical approval:** The research related to human use has been complied with all the relevant national regulations, institutional policies and in accordance with the tenets of the Helsinki Declaration, and has been approved by the authors’ institutional review board or equivalent committee.

### 2.2 Ultrasound examination

**Figure 1:**
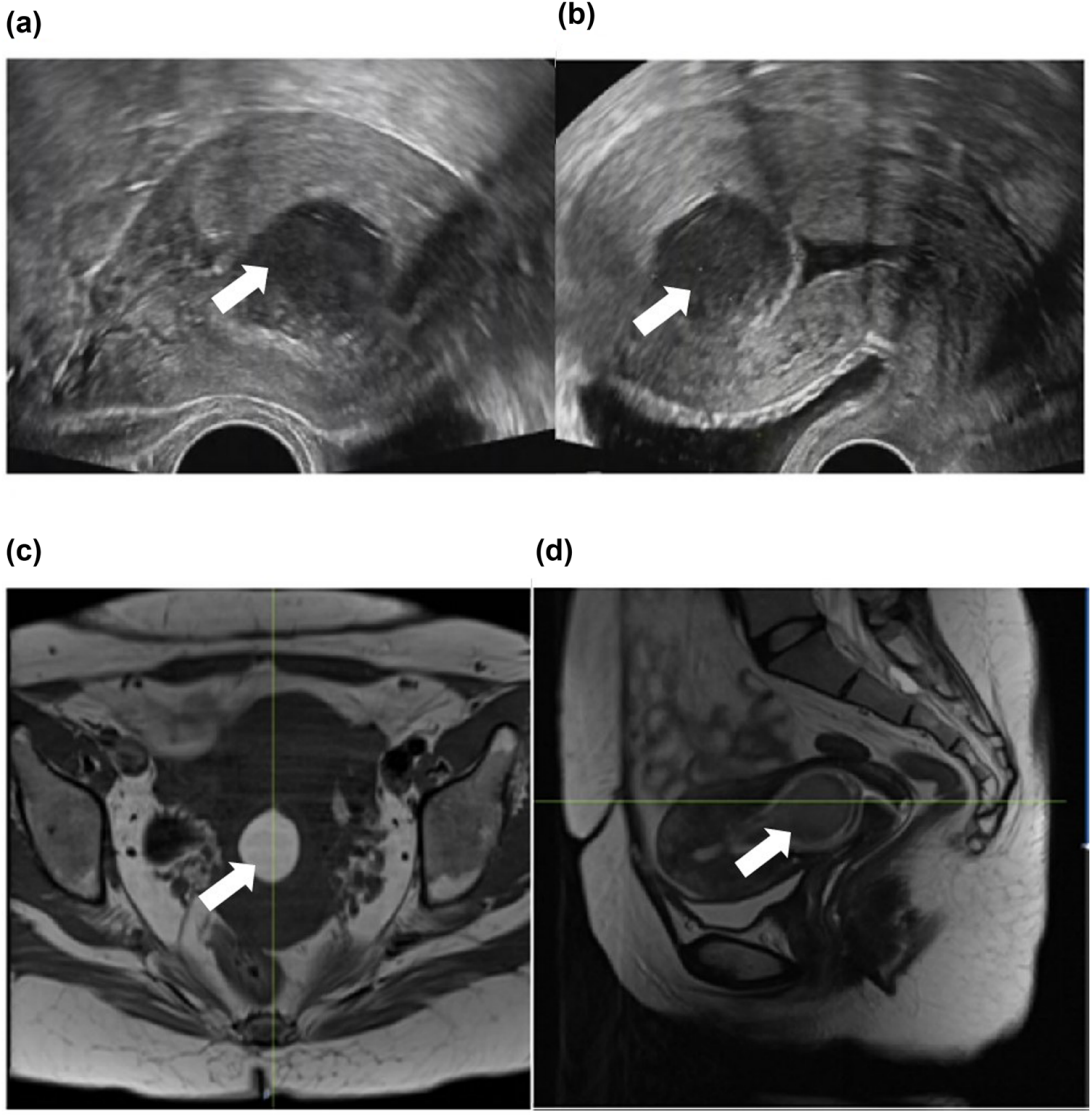
Ultrasonic imaging and MRI of cystic adenomyoma. (a) High signal of lesion in the T1WI cross-sectional images (marked white arrow). (b) Slightly low signal of lesion in the T2WI sagittal images. (c and d) MRI of cystic adenomyoma.

The ultrasound images after admission revealed a cystic mass, 3.0 cm × 2.7 cm in size, which could be seen as a liquid dark area with a dotted linear strong echo and which appeared to be located in the upper part of the uterine cavity. It seemed to form a complete capsule with a thickness of approximately 0.2 cm. The anterior wall of the uterus was not clear, and the lower uterine segment interval was 0.5 cm. The thickness of a single endometrium was approximately 0.1 cm. These results are shown in Figure 1a and b.

### 2.3 MRI examination

The MRI examination after admission showed a circular mass shadow of approximately 2.73 cm × 3.04 cm × 3.61 cm in the endometrial cavity. There was a high signal in the T1-weighted image (WI) and a slightly lower signal in T2WI, and the upper margin of the lesion had a long narrow neck attached to the right anterior myometrium, as shown in Figure 1c and d.

### 2.4 Tumour marker evaluation

Blood samples were collected from the patient on 16 February 2021 at an outpatient review, and enzyme-linked immunosorbent assay results showed that the CA125 concentration was 16.2 U/mL and the CA199 concentration was 285.2 U/mL.

### 2.5 Operation description

**Figure 2:**
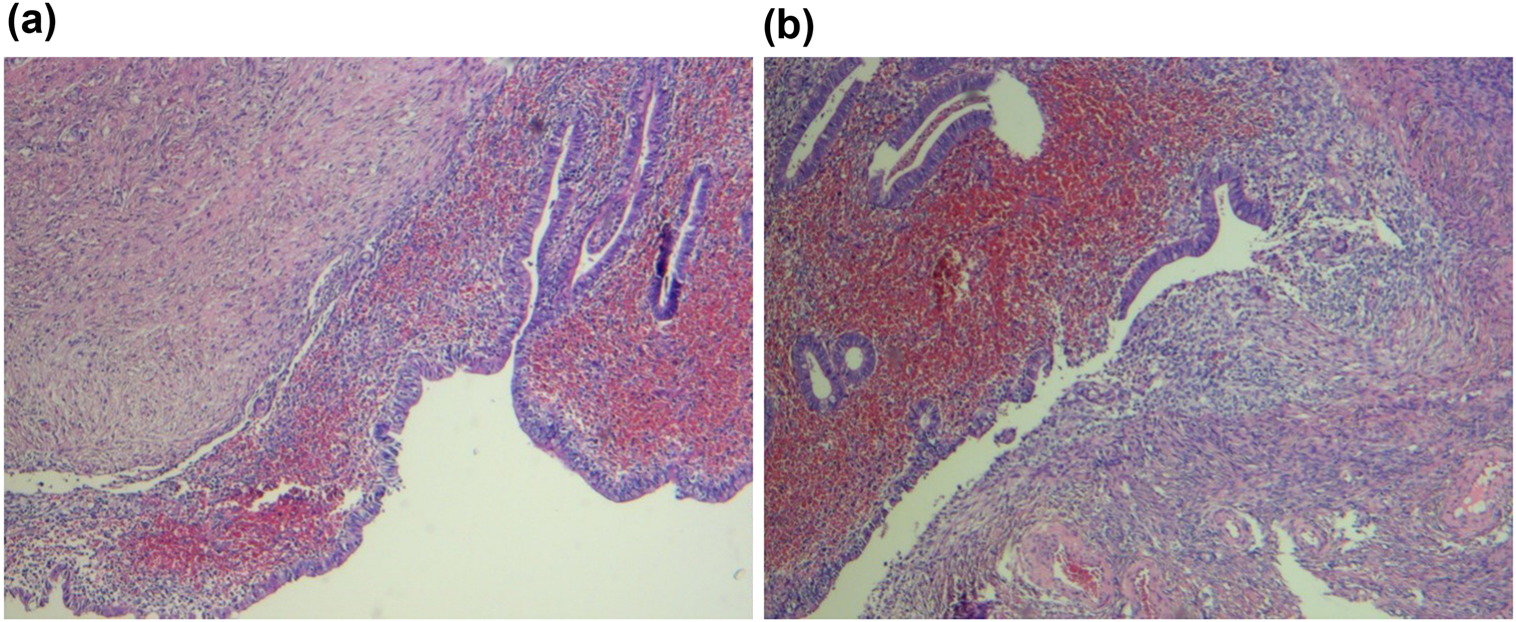
Pathologic examination of the lesion. (a and b) HE staining results of the pathologic lesion.

The preliminary clinical diagnosis was cystic adenomyoma. Hysteroscopy and intrauterine focal electrocision were performed under intravenous anaesthesia. During the operation, a mass of approximately 3 cm × 3 cm × 4 cm was observed to protrude into the uterine cavity from the anterior wall of the uterus. This mass had a smooth surface and was covered in endometrial tissue, and the pedicle, which was approximately 2 cm in width, was attached to the right anterior wall of the uterus, close to the cornual region. Brown chocolate-like fluid exuded from the mass when stimulated with needle electrodes, and the cyst wall was approximately 0.5 cm thick. The hysteroscopy was then performed through the cyst cavity. After washing the cyst cavity, the cyst wall was seen to be smooth and white, and a dilated gland opening was visible near the myometrium cyst wall. A cut was made in the cyst wall using a ring electrode to flush out the region with the normal endometrial tissue while retaining part of the cyst wall to prevent the large area of endometrial defect from affecting postoperative menstruation. The results of postoperative pathology tests suggested the presence of an endometrial cyst as shown in Figure 2. Postoperative intervention comprised the administration of three courses of GnRH-a to prevent relapse. When tested on 2 April 2021, CA199 (21.5 U/mL) had decreased to normal levels, and on 3 July, when menstruation recovered, menstrual abdominal pain symptoms were relieved, and menstrual period and menstrual volume were normal. Informed written consent was obtained from the patient for the publication of this report and any accompanying images.

## 3 Discussion

Diffuse adenomyosis is the most common type, and localised adenomyosis, especially of the cystic uterine glands, is very rare. Cystic adenomyosis is a specific type of lesion that originates in the myometrium of the uterus and is filled with a chocolate-like viscous fluid. A summary of the cases that were found is given in Table 1 [4,5,6,7,8,9,10,11,12,13]. Cystic adenomyoma is characterised by the invasive presence of gland and stromal tissue of the ectopic endometrium with a thickness of greater than 2.5 cm in the myometrium. The main clinical manifestations are secondary progressive dysmenorrhoea, prolonged menstruation, menorrhagia, and uterine enlargement, combined with infertility [14]. Because of the atypical clinical manifestations and the rarity of the condition, patients with cystic adenomyosis are often misdiagnosed. These symptoms may be signs of other clinical conditions, such as subplasmalemmal fibroid degeneration.

**Table 1:** Case summary of cystic adenomyosis reported in literatures

Study (author, time)	Age	Location	Lesion size (cm)	Clinical symptoms	References
Protopapas et al. (2020)	14	Hypotense myometrium	3.8	Dysmenorrhoea	[4]
Takeuchi et al. (2010)	30	Adjacent myometrium	3.5	Serve dyspareunia	[5]
	29	Adjacent myometrium	3	Serve dyspareunia	
	27	Adjacent myometrium	4.2	Serve dyspareunia	
	20	Adjacent myometrium	2.8	Serve dyspareunia	
	30	Adjacent myometrium	3.4	Serve dyspareunia	
	28	Adjacent myometrium	2.5	Serve dyspareunia	
	23	Adjacent myometrium	2.8	Serve dyspareunia	
	20	Adjacent myometrium	3.4	Serve dyspareunia	
Zhou et al. (2020)	45	Posterior uterine isthmus	9	Serve dyspareunia	[6]
Branquinho et al. (2012)	17	Adjacent myometrium	3.3	Dyspareunia	[7]
Dadhwal et al. (2017)	23	Adjacent myometrium	4	Lower abdominal pain, dyspareunia	[8]
	16	Adjacent myometrium	3.5	Lower abdominal pain, dyspareunia	
Kim (2014)	30	Adjacent myometrium	2	Dyspareunia	[9]
Kriplani et al. (2011)	16	Adjacent myometrium	4	Serve dyspareunia	[10]
	18	Adjacent myometrium	5	Serve dyspareunia	
	18	Adjacent myometrium	4.5	Serve dyspareunia	
	24	Adjacent myometrium	4	Serve dyspareunia	
Pontrelli et al. (2015)	27	Uterine cavity	8	Dyspareunia	[11]
Mahey et al. (2023)	20	Left side anteroinferior	3	Lower abdominal pain	[12]
Nigam et al. (2021)	30	Adjacent myometrium	4.8	Severe abdominal pain	[13]

The pathogenesis of cystic adenomyoma is unclear, but it may result from repeated endometrial damage and repair, high oestrogen levels, immunity, inflammation, and other factors [15]. The growth of the endometrial glands and stroma is limited due to the action of oestrogen and progesterone, which causes uniform or limited enlargement of the uterus, and this results in the formation of adenomyoma in the myometrium [16]. The patient in this study had previously undergone a uterine caesarean section, and the wound had healed poorly. She had a history of two abortions and an ectopic pregnancy, and she had had a laparoscopic right salpingectomy. Her history of infection and multiple uterine operations suggests probable disruption of the boundary between the endometrium and the myometrium, and the endometrium migration to the myometrium is a known high-risk factor for the development of the disease.

The surgical techniques described for treating cystic adenomyomas were developed from myomectomy techniques [3]. Individualised treatment plans should be formulated according to the patient’s age of onset of the disease, lesion location, size, and fertility requirements and should be aimed at relieving symptoms, removing lesions, protecting fertility function, and avoiding recurrence. Panhysterectomy and lesion resection may be opted for depending on the patient’s age and fertility requirements, and lesion resection is the preferred surgical method for treating young patients who may wish to have a child in the future.

There is no unified conclusion concerning the necessity and choice of maintenance treatment after surgery. Some scholars regard cystic adenomyoma to be a special type of adenomyosis [17]. They did not think that it needs maintenance and the patients can have a treatment efficiency; however, in this case, the postoperative pathology outcome was an endometrioma, and, due to the probable presence of residual ectopic endometrial tissue after surgery, the postoperative use of drugs to prevent recurrence was advocated. Since part of the cyst wall was preserved to replace the normal endometrium, three courses of GnRH-a were prescribed.

## 4 Conclusion

According to the current case and a review of the relevant literature, cases of cystic adenomyoma are clinically significant. For women with aggravated menstruation, severe dysmenorrhoea, isolated uterine cysts seen in ultrasound images, and elevated serum CA125, the possibility of cystic uterine adenomyomas should be considered to avoid clinical misdiagnosis and delays in the diagnosis of the condition. In such cases, MRI scans facilitate further differential diagnosis, and surgical treatment is the most effective treatment method. Future studies are required to gain insights into maintenance treatment after surgery.
